# A Combined *In Vivo*, *In Vitro*, *In Silico* Approach for Patient-Specific Haemodynamic Studies of Aortic Dissection

**DOI:** 10.1007/s10439-020-02603-z

**Published:** 2020-09-14

**Authors:** Mirko Bonfanti, Gaia Franzetti, Shervanthi Homer-Vanniasinkam, Vanessa Díaz-Zuccarini, Stavroula Balabani

**Affiliations:** 1 Wellcome/EPSRC Centre for Interventional and Surgical Sciences (WEISS), Department of Medical Physics and Biomedical Engineering, University College London, 43-45 Foley Street, London, W1W 7TS UK; 2 Department of Mechanical Engineering, University College London, Torrington Place, London, WC1E 7JE UK; 3 Leeds Teaching Hospitals NHS Trust, Great George Street, Leeds, LS1 3EX UK

**Keywords:** Aortic dissection, Particle image velocimetry, Blood flow, Computational fluid dynamics, Pulsatile flow, Patient-specific

## Abstract

The optimal treatment of Type-B aortic dissection (AD) is still a subject of debate, with up to 50% of the cases developing late-term complications requiring invasive intervention. A better understanding of the patient-specific haemodynamic features of AD can provide useful insights on disease progression and support clinical management. In this work, a novel *in vitro* and *in silico* framework to perform personalised studies of AD, informed by non-invasive clinical data, is presented. A Type-B AD was investigated *in silico* using computational fluid dynamics (CFD) and *in vitro* by means of a state-of-the-art mock circulatory loop and particle image velocimetry (PIV). Both models not only reproduced the anatomical features of the patient, but also imposed physiologically-accurate and personalised boundary conditions. Experimental flow rate and pressure waveforms, as well as detailed velocity fields acquired *via* PIV, are extensively compared against numerical predictions at different locations in the aorta, showing excellent agreement. This work demonstrates how experimental and numerical tools can be developed in synergy to accurately reproduce patient-specific AD blood flow. The combined platform presented herein constitutes a powerful tool for advanced haemodynamic studies for a range of vascular conditions, allowing not only the validation of CFD models, but also clinical decision support, surgical planning as well as medical device innovation.

## Introduction

Aortic dissection (AD) is a life-threatening vascular condition in which an intramural tear results in blood flowing within the medial layer of the aortic wall leading to the development of an intimal flap (IF). The IF separates the true lumen (TL), the physiological pathway of blood, from a false lumen (FL), the new pathological route within the aortic wall.

The optimal treatment of Type-B dissections—those involving the arch and descending aorta—is still debated; when uncomplicated, they are commonly managed medically, but up to 50% of the cases will develop complications requiring invasive intervention. Currently, some anatomical predictors of AD growth are used to customise the follow-up and treatment planning; however, haemodynamic information such as flow patterns, pressures, velocity and wall shear stress (WSS) indices can provide a more comprehensive understanding of the condition, adding prognostic value.^11^

There is a great amount of evidence that haemodynamics plays an important role in AD progression. For example, high intra-luminal pressure and pressure imbalance between the lumina are key drivers of FL dilation and distal extension of the dissection, while WSS greatly affects aortic remodelling.^28^ Elevated time-averaged WSS (TAWSS) has been associated with retrograde Type-A dissections and tear initiation,^19^ while low and oscillatory WSS and vortical flow have been correlated with FL shrinkage and thrombosis.^29^ However, accurate haemodynamic measurements are difficult to obtain non-invasively *in vivo*.

In the last decade, personalised computational fluid dynamics (CFD) models have been investigated as a tool to improve the understanding and clinical outcome of AD.^13^ Advances in modelling and simulation have allowed for increasingly realistic AD models. Geometrical accuracy has improved from the representation of only the dissected aorta with the upper branches^9^ to the inclusion of the abdominal and visceral vessels.^21^ Simplified ‘static’ outlet boundary conditions (BCs), such as constant pressure or prescribed flow splits, have been replaced by more physiologically-accurate ‘dynamic’ three-element Windkessel models (3WKs).^12^ Recently, the limitation of the rigid wall assumption has also been overcome, with the implementation of fluid structure interaction (FSI) models able to simulate the motion of the aortic wall and IF.^1^,^2^,^4^

Experimental investigations of flow in simplified models of AD have increased the general understanding of the pathology and highlighted the influence of morphological features on the involved fluid dynamic variables.^3^,^23^ However, patient-specific, complex flow fields have not been measured in physical AD models yet. Currently, there are no AD experimental models able to accurately reproduce personalised haemodynamics conditions, by using both anatomically-accurate phantoms and physiologically-correct BCs.

Complementing CFD simulations with experimental flow measurements can allow extensive validation of computational results, increasing the confidence in the numerical predictions. Validation of *patient-specific* CFD simulations of AD is currently based on *in vivo*, 2D and 4D phase-contrast magnetic resonance imaging (PC-MRI) data.^21^ However, these modalities, when used in a clinical setting, have poor resolution and are often subject to noise. Few studies have attempted to combine *in vitro* and *in silico* approaches for the study of AD hitherto. Chen *et al.*^8^ developed a 3D FSI model of an idealised AD and validated the results against *in vitro* ultrasound measurements in a porcine AD model in a pulse duplicator system; however, clinical ultrasound measurements exhibit limited resolution. An established flow diagnostics technique, particle image velocity (PIV), can provide high temporal and spatial resolution velocity fields for visualisation and quantification of vascular flows. The use of an experimental technique, such as PIV, allows the validation of numerical models in a highly-controlled environment, with a level of reproducibility and accuracy not possible *in vivo*.^22^ PIV was recently used to study the impact of tear size on the flow in an idealised model of AD using computations and experiments.^30^ However, to date, PIV has not been used in any patient-specific *in vitro* AD work.

The main aim of this work is to develop a combined *in vitro*, *in silico* approach for the study of the patient-specific haemodynamics of AD informed by non-invasive *in vivo* data. This work presents several novel aspects:

1. For the first time an experimental mock circulatory loop for AD investigations was developed alongside state-of-the-art CFD simulations, that includes not only an anatomically-accurate phantom, but also physiologically-accurate and personalised BCs.
2. This allowed the study of a Type-B AD with both numerical and experimental tools, informed by clinical data to accurately reproduce patient-specific haemodynamic conditions.
3. Experimental PIV acquisitions, alongside pressure and flow rate measurements, allowed an extensive comparison between measured and predicted haemodynamic parameters and the validation of the CFD model.
4. A new paradigm to guide clinical decisions is introduced.

## Materials and Methods

**Figure 1:**
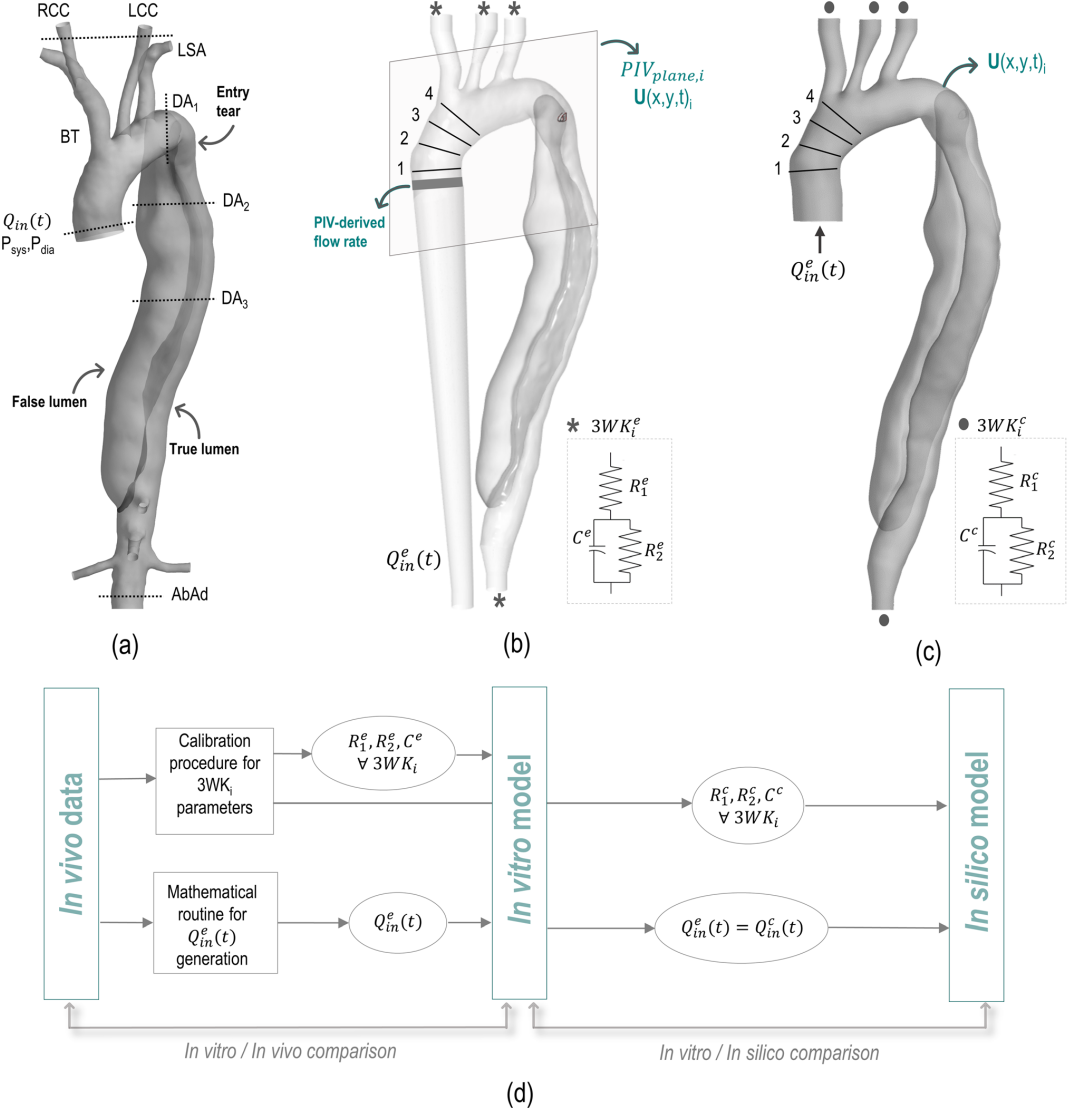
(a) Geometry of the dissected aorta as extracted from the CT scan. The dotted lines indicate the sections where 2D PC-MRI data was acquired. (b) *In vitro* phantom with physical boundary conditions. (c) *In silico* 3D fluid domain and corresponding boundary conditions. (d) Schematic of the *in vivo*, *in vitro* and *in silico* approach developed and implemented in this work. Clinical non-invasive data (2D PC-MRI derived flow rate and brachiocephalic pressure) is used to inform both the experimental and computational models. *In vitro* results are compared with *in vivo* data to assess that the fluid dynamic conditions meet the patient-specific criteria, while *in silico* results are compared with *in vitro* measurements to validate the computational simulation. The position of the four lines where velocity profiles have been extracted from both PIV data and CFD results for comparison purposes are shown in (b) and (c).

Figure 1 provides an overview of the *in vivo* - *in vitro* - *in silico* approach developed in this work, which is detailed in the following sections.

### Clinical Data and Vessel Segmentation

The study is based on a clinical dataset of a 77-year-old male subject with a chronic Type-B AD. The dataset was acquired as part of an ethically-approved protocol at the Leeds General Infirmary (NHS Health Research Authority, ref: 12/YH/0551; Leeds Teaching Hospitals NHS Trust, ref: 788/RADRES/16) and consent was obtained from the patient.

The dataset included a computed tomography (CT) scan of the entire aorta and 2D PC-MRI scans at several locations of the aorta and upper branches, as indicated in Fig. 1a. Systolic and diastolic brachial pressure values were also acquired with a sphygmomanometer. Flow information was extracted from the PC-MRI sequences using the software GTFlow (GyroTools, Switzerland), while the AD geometry was segmented from the CT scan with Simpleware ScanIP (Synopsys, USA). Full details on the image acquisition and segmentation procedures are reported in Bonfanti *et al*.^5^

The dissection originated distal to the left subclavian artery (LSA) and extended to the celial trunk. Only one entry tear located approximately 10 mm distal to the proximal end of the dissection was evident from the clinical images, while no re-entry tears were detected (Fig. 1a).

### Experimental Setup

A 3D phantom of the patient-specific AD was manufactured by 3D printing technology (Materialise NV, Belgium) to obtain a rigid, transparent model (Fig. 1b).

The abdominal aortic branches were excluded from the phantom, which terminated just after the distal end of the dissection, in order to simplify the geometry of interest. Connectors were added to the inlet and brachiocephalic trunk (BT), left common carotid (LCC), LSA, and descending aorta (DA) outlets of the phantom to facilitate the connection to the experimental setup.

**Figure 2:**
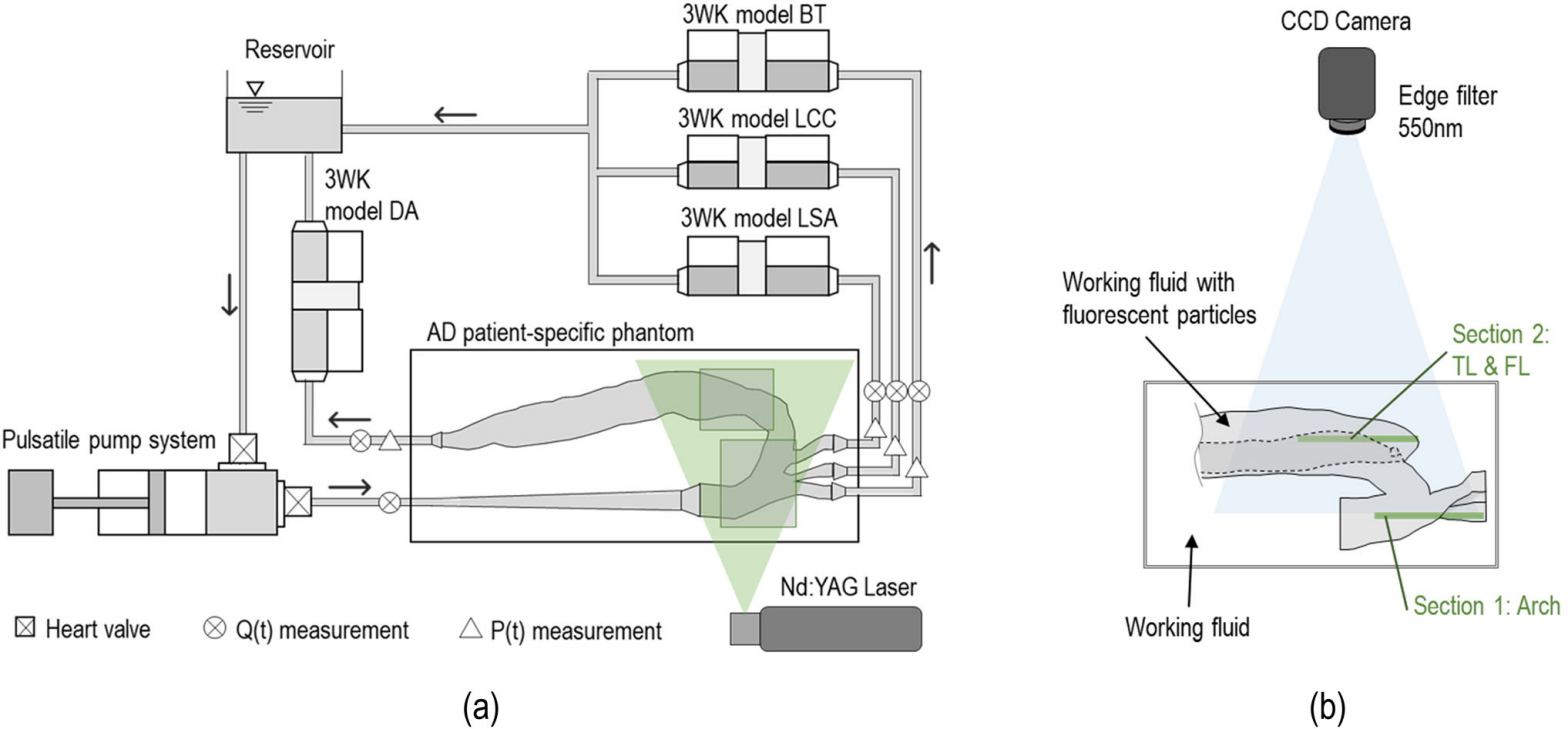
(a) Schematic illustration of the experimental platform highlighting the positions where flow rate and pressure curves were acquired and PIV was performed. (b) Detail of the aortic phantom showing the imaged sections for the PIV acquisitions.

A pulsatile flow circuit was developed^14^ comprising a computer-controlled piston pump and left ventricle simulator, the AD phantom, a tunable 3WK model at each aortic outlet and an atrial reservoir. A schematic of the experimental arrangement used is shown in Fig. 2. A detailed description of the components can be found in Franzetti *et al.*^14^ Pressure (P) and flow rate (Q) waves were acquired in real time using pressure transducers (Omega Engineering, UK) and an ultrasound flow meter (Sonotec, Germany), respectively, at the inlet and outlets of the phantom, as indicated in Fig 2a. The signals were acquired *via* a custom made LabVIEW virtual instrument using a CompactRIO controller (cRIO-9040, 1.3 GHz Dual-Core, 70T, FPGA, RT, 4-Slot; National Instruments, USA) with a sampling sequence of 200 Hz. A Savitzky-Golay low-pass filter was employed to attenuate high frequencies in the pressure signals. The experimental platform was tuned in order to reproduce patient-specific conditions using the personalisation procedures described in Section "Model Personalisation".

For PIV measurements, the flow was seeded with fluorescent polymer particles (PMMA-RhB-10, 1–20 *μ*m ; Dantec Dynamics, Denmark) with a mean diameter of 10 *μ*m, injected into the flow upstream of the phantom and allowed to disperse uniformly within the aortic model.

The tracing particles were illuminated by a pulsed Nd:YAG laser (Litron Lasers, Bernoulli, UK) emitting @ 532 nm wavelength light. Pairs of particle images were recorded with a charge-coupled device camera (Imperx, USA) at a sampling rate of 22 Hz with a resolution of 4000 $\times$ 3000 pixels and a time interval of 1 ms. An optical cut-off filter @ 550 nm was used to allow only the particles’ fluorescent signal through the camera.

Two separate measurements were taken to acquire the flow field in the aortic arch—section 1—and in the proximal part of the TL and FL in the descending aorta—section 2—as shown in Fig. 2b.

Image acquisition was performed using the TSI Insight4G software (TSI, USA), that was also used to synchronise the camera and the laser pulses *via* a Laser-Pulse synchroniser (TSI, Model 610006).

The acquired images were subsequently processed in PIVlab.^27^ Velocity fields were generated using the fast Fourier transform based cross-correlation algorithm, implemented with a three-pass technique, starting with an interrogation area of 64 $\times$ 64 pixels and ending with an area of 32 $\times$ 32 pixels, overlapping by 50%. The normalised median test was applied after each pass, evaluating the velocity fluctuations with respect to the median value in a 5 $\times$ 5 neighbourhood around the vector. Lastly, a smoothing filter was applied to the velocity fields after each iteration in order to decrease the amount of noise introduced by the algorithm and increase the quality of the velocity estimation.^22^ Post-processing was performed to extract the parameters of interest with custom developed MATLAB (MathWorks, USA) routines.

Phase-averaged velocity fields $\langle \mathbf{u} (x,y,t) \rangle$ were extracted from the instantaneous velocity data $\mathbf{u} (x,y,t)$. Convergence was evaluated by plotting the phase-averaged velocity in small regions as a function of the number of cycles considered; 10 cardiac cycles were found to be sufficient to reach converged average values with variation $<1.6\%$.

In order to quantify the error in the velocity measurements, the flow rate was calculated from the phase-averaged velocity field at 28 successive sections of the aortic inlet (grey area highlighted in Fig. 1b) by performing double integration under the assumption of axial-symmetrical flow. Using the mass conservation principle, the difference between the flow rate values led to an error of 5.32%.^26^

The working fluid was selected in order to match the refractive index (RI) of the phantom material as closely as possible $(RI = 1.50 - 1.51)$. In the present study, a potassium thiocyanate (KSCN) water solution (63% by weight) was selected as a Newtonian blood mimicking fluid as in previous works.^15^,^18^ Since it was not possible to create a test fluid with the same viscosity and density of blood while matching the RI of the phantom wall, a compromise had to be made in accepting a higher fluid density ($\rho$ = 1310 Kg/m^3^) and a lower viscosity $(\upmu = 2.2 \; {\text{cP}})$. However, the nondimensional parameters characterising the experimental flow remained in the physiological range (see Section "Numerical Simulations"). The impact of this approximation was further investigated with the *in silico* model, as reported in Section "Limitations".

### Model Personalisation

The experimental and numerical BCs were tuned to reproduce the patient under investigation as follows.

The protocol described in Franzetti *et al.*^14^ was adopted to obtain the analytical waveform reproducing as closely as possible the aortic flow rate of the patient, which was measured with 2D PC-MRI. First, the main physiological parameters were extracted from the *in vivo* waveform (i.e. stroke volume (SV) = 107.6 mL, cycle length (T) = 0.8 s and mean flow rate = 134 mL/s). Second, the parameters were adjusted for compatibility with the image acquisition rate $(f = 22\,\hbox {Hz})$; in particular, T was increased to 0.82 s to be a multiple of 1/*f* whilst maintaining the same SV and mean flow rate. Finally, the correct analytical waveform was generated through a computer routine.

The parameters of the 3WK models were selected following the procedure described in Bonfanti *et al.*^5^ with the aim to obtain the patient-specific systolic and diastolic pressure values and correct cardiac output (CO) distribution amongst the aortic branches. The *target* values for the calibration procedure are listed in Table 1.

The estimated resistance (*R*) and compliance (*C*) values were used to tune the physical components of the experimental rig^14^ and to set the outflow BCs of the numerical model, described in the following section. The set experimental and computational 3WK values differ slightly due to the latter including the hydraulic resistance contribution of connectors and tubes between the phantom and the physical 3WK, which were not included in the 3D computational domain. Such discrepancy is although negligible. The values of the 3WK parameters used in the numerical model are listed in Table 2.

**Table 1:** Systolic $(P_{\text {sys}})$ and diastolic $(P_{\text{dia}})$ blood pressure values and mean flow rate $(\bar{Q})$ at the aortic branches used as target values for the personalisation procedure.

Parameter	Value	Unit	Source
$P_{\text {sys}}$	150	mmHg	Sphygmomanometer
$P_{\text{dia}}$	80	mmHg	Sphygmomanometer
$\bar{Q}_{\text{IN}}$	134.5	mL/s	2D PC-MRI
$\bar{Q}_{\text{BT}}$	22.5	mL/s	2D PC-MRI^a^
$\bar{Q}_{\text{LCC}}$	8.9	mL/s	2D PC-MRI
$\bar{Q}_{\text{LSA}}$	9.8	mL/s	2D PC-MRI^a^
$\bar{Q}_{\text{DA}}$	93.3	mL/s	2D PC-MRI

^a^The value of these parameters were calculated based on patient-specific 2D PC-MRI data and physiological considerations as explained in Bonfanti *et al.*^4^

**Table 2:** Parameters of the three-element Windkessel models (3WK) coupled to the outlets of the numerical aortic model.

3WK	*R*_1_ [mmHg s mL^−1^]	*R*_2_ [mmHg s mL^−1^]	*C* [mL mmHg^−1^]
BT	0.25	6.00	0.30
LCC	0.63	10.00	0.07
LSA	0.57	10.00	0.07
DA	0.01	1.15	0.30

### Numerical Simulations

The Navier–Stokes and continuity equations for 3D time-dependent flows were solved with the CFD solver ANSYS-CFX 19.0 (ANSYS, USA). The equations were spatially and temporally discretised with a high-resolution advection scheme and a second order implicit backward Euler scheme, respectively, using a uniform time-step of 1 ms, small enough for time-step size-independent results. The fluid was treated as Newtonian and incompressible with $\mu$ and $\rho$ matching those of the KSCN solution used in the experiments, as reported in Section “Experimental Setup”.

The *in silico* 3D fluid domain comprised the lumen of the aortic phantom (Fig. 1c). The BCs adopted in the CFD model are shown schematically in Fig. 1c. The experimental flow rate waveform was prescribed at the inlet with a flat velocity profile. WK3s were coupled to the outlets with parameters listed in Table 2. The 3D model walls were assumed rigid with a no-slip BC, to reproduce the *in vitro* phantom.

Based on the inlet cross-sectional area of the aorta and the inlet flow rate, the mean $(Re_{\text{m}})$ and peak $(Re_{\text{p}})$ Reynolds numbers were equal to 3472 and 11581, respectively, while the Womersley number (*Wo*) was 32. Since $Re_{\text{p}}$ was higher than the critical Reynolds number $(Re_{\text{c}})$ for transition to turbulent flow (calculated following Kousera *et al.*^17^ as $Re_{\text{c}} = 250 \times Wo$), the Shear Stress Transport (SST) turbulence model was employed in the simulation. The SST model is a Reynolds-Averaged Navier-Stokes (RANS) approach which combines the $k-\omega$ model for the inner region of the boundary layer with the $k-\epsilon$ model for the outer region, and it is commonly used in CFD models of AD flows.^1^ An inflow turbulence intensity (Tu) equal to 1% was applied, in agreement with the findings of Kousera *et al.*^17^ The sensitivity of the results to this parameter was tested by also running a simulation with a Tu of 5%; the comparison against the results obtained with a Tu of 1% showed only minor changes in the calculated velocity field (maximum and mean difference in the fluid domain at peak systole equal to 7.3 and 0.1%, respectively). The fluid domain was discretised with Fluent meshing (ANSYS), adopting a tetrahedral grid in the core region and ten prism layers at the walls. The dimensionless height (*y*+) of the cell adjacent to the wall was kept less than 2 for an accurate resolution of the near-wall velocity profile with the employed turbulence model. A sensitivity analysis was performed to guarantee the independence of the results to the mesh. Three computational meshes of 2.3M, 4.1M and 6.1M cells were tested. Comparison of numerical results showed that the relative difference in terms of peak velocity, pressure and flow rate at the boundaries were less than 2.0, 1.1 and 1.9%, respectively, between the coarse and medium mesh, and less than 0.5, 0.1 and 0.5%, respectively, between the medium and fine mesh. Therefore, the medium mesh with 4.1M elements was selected for the analysis. Simulations were run for three cycles in order to reach the periodic steady-state on the high-performance computing cluster of UCL Computer Science Department (computational time: 11 h/cycle). The convergence of the solution was controlled by specifying a maximum root-mean square residual of 10$^{-5}$. Post-processing was performed using CFD-Post (ANSYS) and MATLAB.

### *In Vivo*, *In Vitro*, *In Silico* Comparison

Experimental and numerical P and Q waveforms were first compared to the clinical systolic and diastolic pressures and patient-specific CO distribution to verify that the models met the *in vivo* condition. Then, AD haemodynamics was analysed using the *in vitro* and *in silico* aortic velocity fields in two sections of interest (i.e. Sections 1 and 2 shown in Fig. 2b). The aortic arch and proximal part of TL and FL—where the tear is located—were selected as deemed more appropriate to illustrate our approach in this patient-specific case of AD. A qualitative comparison was performed between the in-plane velocity magnitudes. Quantitatively, computational and experimental velocity profiles over the four lines shown in Figs. 1b and 1c were extracted by calculating the in-plane axial velocity (i.e. normal to the section line, $u_n$) at four instants of the cardiac cycle (i.e. acceleration, peak systole, deceleration and diastole). The percentage difference $(\Delta )$ between the experimental and computational profiles was calculated as

1 $$\begin{aligned} \Delta = \frac{1}{N}\sum _{i=1}^{N} \frac{\left| u_{n,i}^{e}-u_{n,i}^{c}\right| }{{\text{max}}_j \left| u_{n,j}^{c}\right| } \end{aligned}$$

where $u_{n,i}^{e}$ and $u_{n,i}^{c}$ are the experimental and computational velocity profiles, respectively, and *N* is the total number of data points per line (in this case equal to 1000).

## Results

### Flow Rate and Pressures

**Figure 3:**
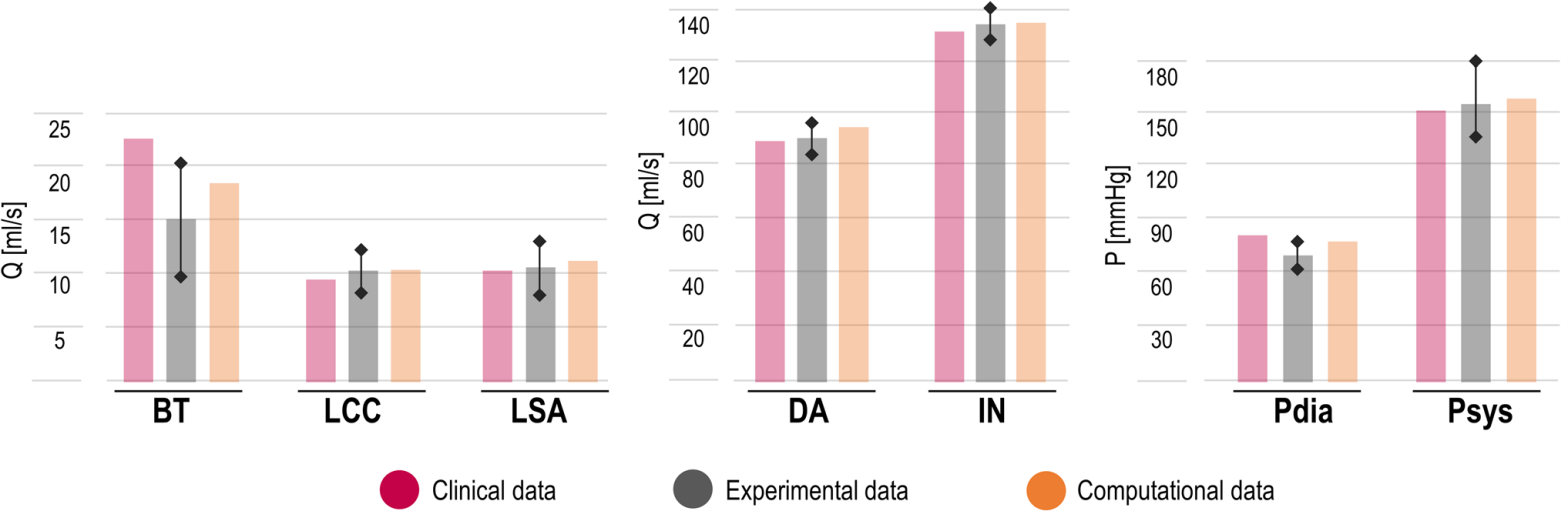
Comparison between mean flow rate (*Q*) values at the aortic outlets and systolic ($P_{\text {sys}}$) and diastolic ($P_{\text{dia}}$) pressures at the inlet obtained with the experimental and computational models against the target clinical values. Error bars are reported for the experimental data, which include the standard deviation (SD) due to inter-cycle variability as well as the amplitude of the experimental oscillations due to the mechanical action of the aortic valve for the systolic pressure value.

The personalisation procedures employed to calibrate the model BCs allowed the reproduction of the *in vivo* condition. The experimental inlet flow rate was characterised by *T* = 0.82 s, corresponding to a heart frequency of 73 bpm, mean flow rate of 137.4 ± 5.4 mL/s, and SV of 106 ± 4.4 mL, closely matching the parameters extracted from the 2D PC-MRI. Comparisons between target clinical pressure and mean flow rate values, and experimental and computational results are shown *via* histograms in Fig. 3. The clinical systolic/diastolic pressure values and mean flow rate at the outlets are within the experimental range with the only exception of BT, where the clinical value exceeds the upper experimental bound by 1.5 mL/s, equivalent to an error of 6%. These results indicate that the personalisation procedure of the experimental platform was successful in reproducing the patient-specific haemodynamic features of the case under investigation. The obtained computational predictions compare well with the experimental results, meeting the calibration targets (Fig. 3).

**Figure 4:**
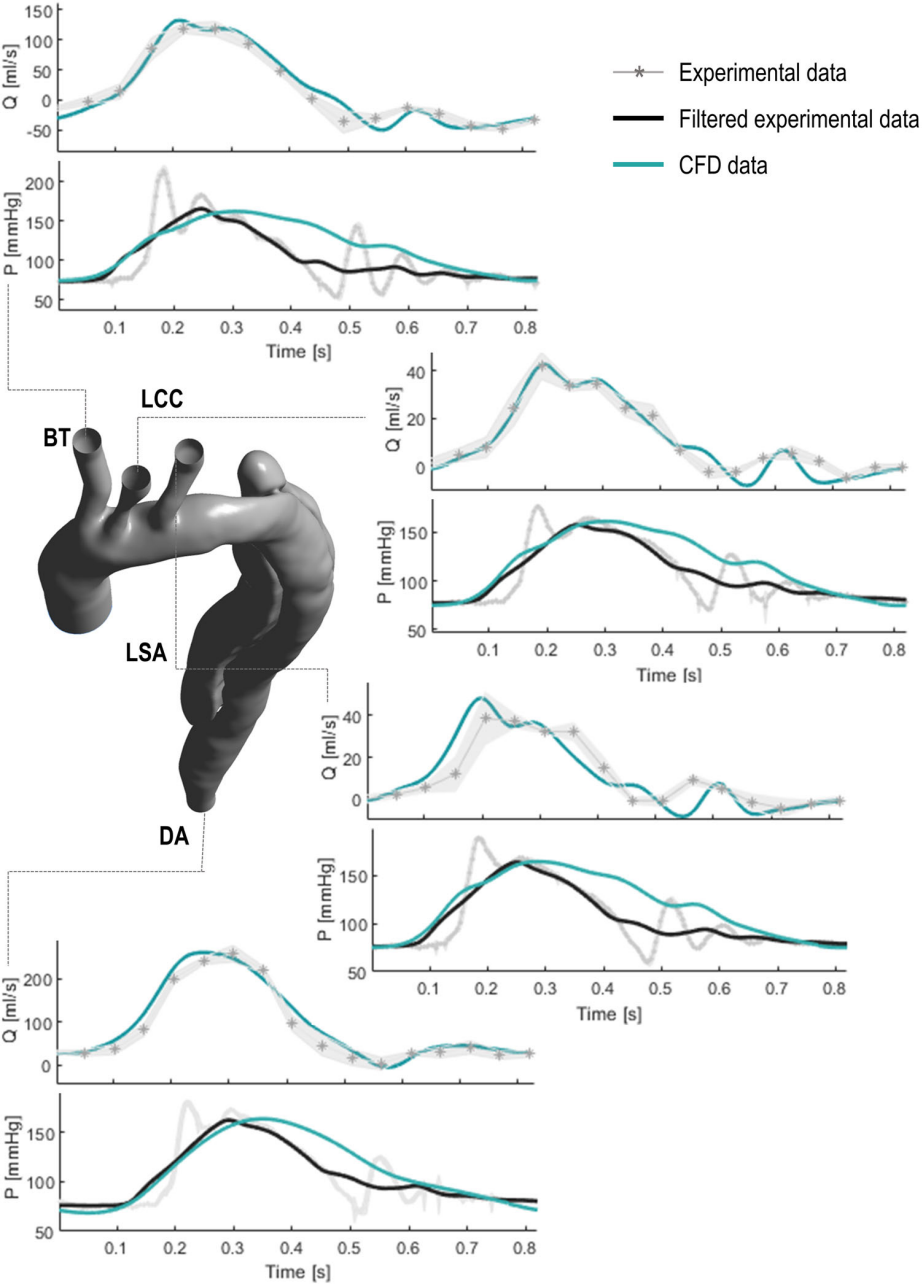
Comparison between flow rate (*Q*) and pressure (*P*) waveforms obtained from numerical and experimental results at the outlets of the aortic domain. The experimental data is reported with standard deviation (SD) intervals. The filtered experimental pressure waveforms are also shown to facilitate the comparison against the computational results.

Flow rate and pressure waveforms acquired experimentally at the four outlets of the phantom are compared against the corresponding numerical results in Fig. 4. Good agreement is found between the *in vitro* and *in silico* results, both in terms of values and shape of the waveforms.

The oscillations observed in the measured pressure waveforms—whose amplitude decreases along the flow direction—are a consequence of the mechanical valve and correspond to its opening and closing phases at the beginning of systole and diastole, respectively. The filtered experimental curves were considered in the quantitative comparison against the results of the computational model, since the latter did not include the action of the mechanical valve. As shown in Fig. 4, numerical predicted diastolic pressure values are systematically higher than those measured experimentally. The rate of decay of the diastolic pressure is described by the time constant, or decay time $(\tau = RC)$, characterising the 3WKs coupled to the aortic outlets. Lower values of $\tau$ are observed in the experiments when compared to the simulations, which may be explained by smaller *R* and/or *C* values in the physical 3WKs with respect to the nominal ones. However, the pressure range observed *in vitro* and *in silico* is very similar as indicated by the very good agreement between the minimum and maximum pressure values, with the highest difference of − 2.3% for the peak value at LCC, and 10.7% for the minimum diastolic value at DA (Fig. 4).

### Velocity Fields and Profiles

**Figure 5:**
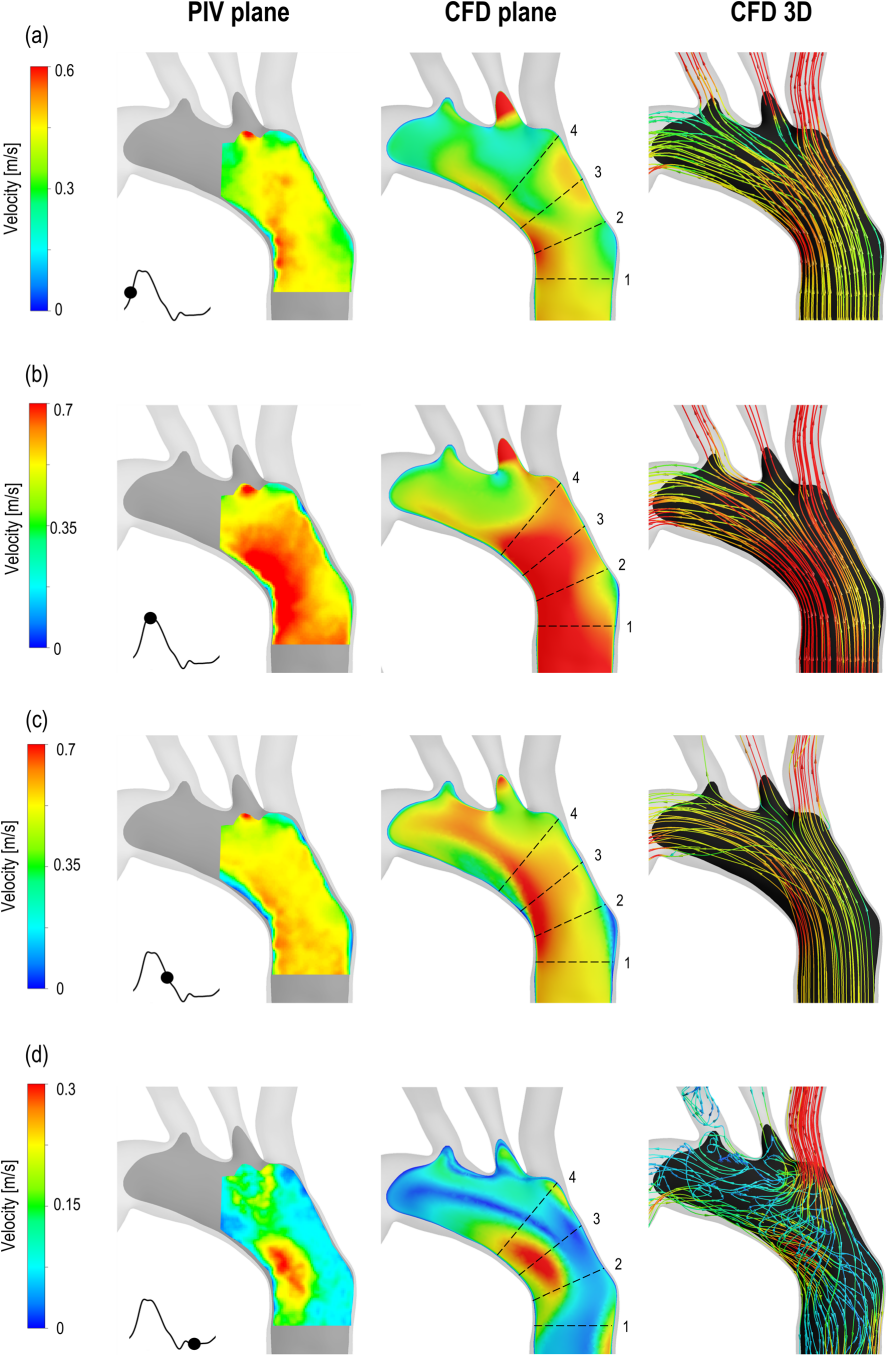
Comparison between experimental PIV-derived phase-averaged velocity magnitude fields (left column) and corresponding numerical predictions (middle column) in the aortic arch. The 3D streamlines obtained with the CFD simulation is reported in the right column. The comparison is shown for four different instants of the cycle: (a) acceleration, (b) peak systole, (c) deceleration and (d) diastole. The dashed lines indicate the position for the extraction of the velocity profiles reported in Fig. 6.

The velocity fields in the ascending aorta and aortic arch obtained by numerical simulations and PIV measurements are presented in Fig. 5. The results show the flow development during a cardiac cycle. Due to optical constraints and light refraction, the PIV-derived phase-averaged velocities are restricted to a region of the imaged plane. Highly organised motion is observed during the acceleration and systolic phases (Figs. 5a and 5b) reaching a maximum velocity of 0.95 and 0.74 m/s towards the inner wall of the aortic arch for CFD and PIV, respectively. A flow separation region at the inner curvature of the aorta forms during the deceleration phase (Fig. 5c), while disorganised streamlines characterise the flow in the aortic arch during diastole, with velocity values up to 0.31 and 0.28 m/s for CFD and PIV respectively. The 3D nature of the flow is illustrated by the 3D streamlines obtained with the computational model (Fig. 5, right column): ordered streamlines can be observed during the systolic phase in the ascending aorta, where the fluid flows in parallel layers, while disturbed, 3D flow is evident in diastole. The numerical and experimental velocity distributions are qualitatively and quantitatively similar throughout the cardiac cycle. Slight discrepancies can be observed in diastole (Fig. 5d) where experimental error is expected to be higher.

**Figure 6:**
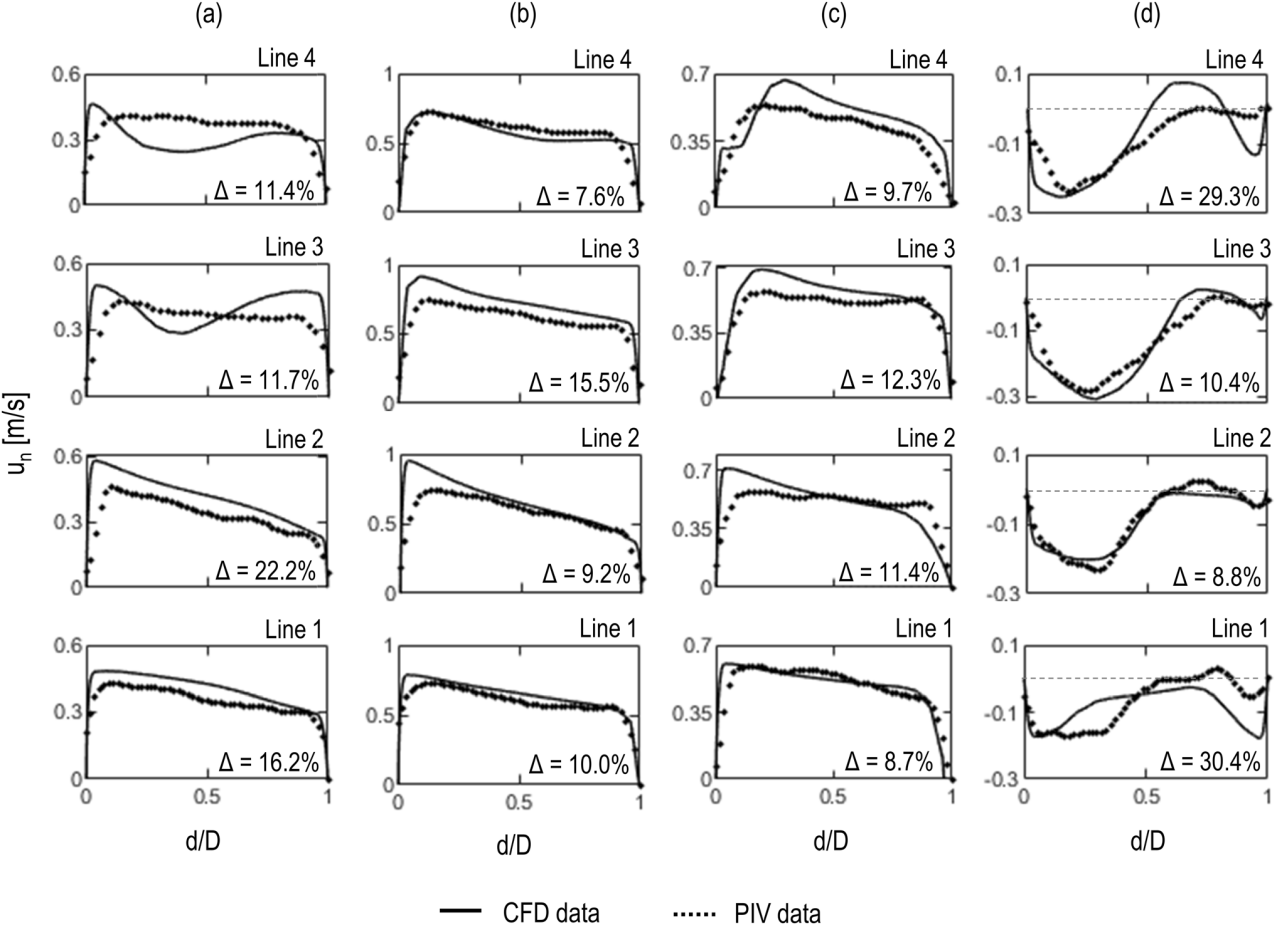
Experimental and numerical axial velocity profiles $(u_n)$ at (a) acceleration, (b) peak systole, (c) deceleration and (d) early diastole. For the position of lines 1–4 refer to Fig. 5. The average percentage difference $(\Delta )$ between the two curves calculated according to Eq. (1) is reported in the bottom-right corner of the plots.

A closer comparison between the experimental and computational results is provided in Fig. 6 by plotting axial velocity profiles $(u_n)$ over the four selected lines shown in Fig. 5. The predicted and measured velocities agree remarkably well. Skewed velocity profiles can be observed during systole (Fig. 6b), with highest velocity values towards the inner wall of the arch, while bidirectional velocity profiles are evident at early diastole (Fig. 6d), characterised by retrograde flow along the inner wall and anterograde flow along the outer wall of the arch.

The percentage difference between the experimental and computational profiles are reported in Fig. 6. A maximum difference of 30% is noted at early diastole for the profile in line 1 which can be attributed to the lower velocity values and associated PIV errors, as discussed in the following section.

**Figure 7:**
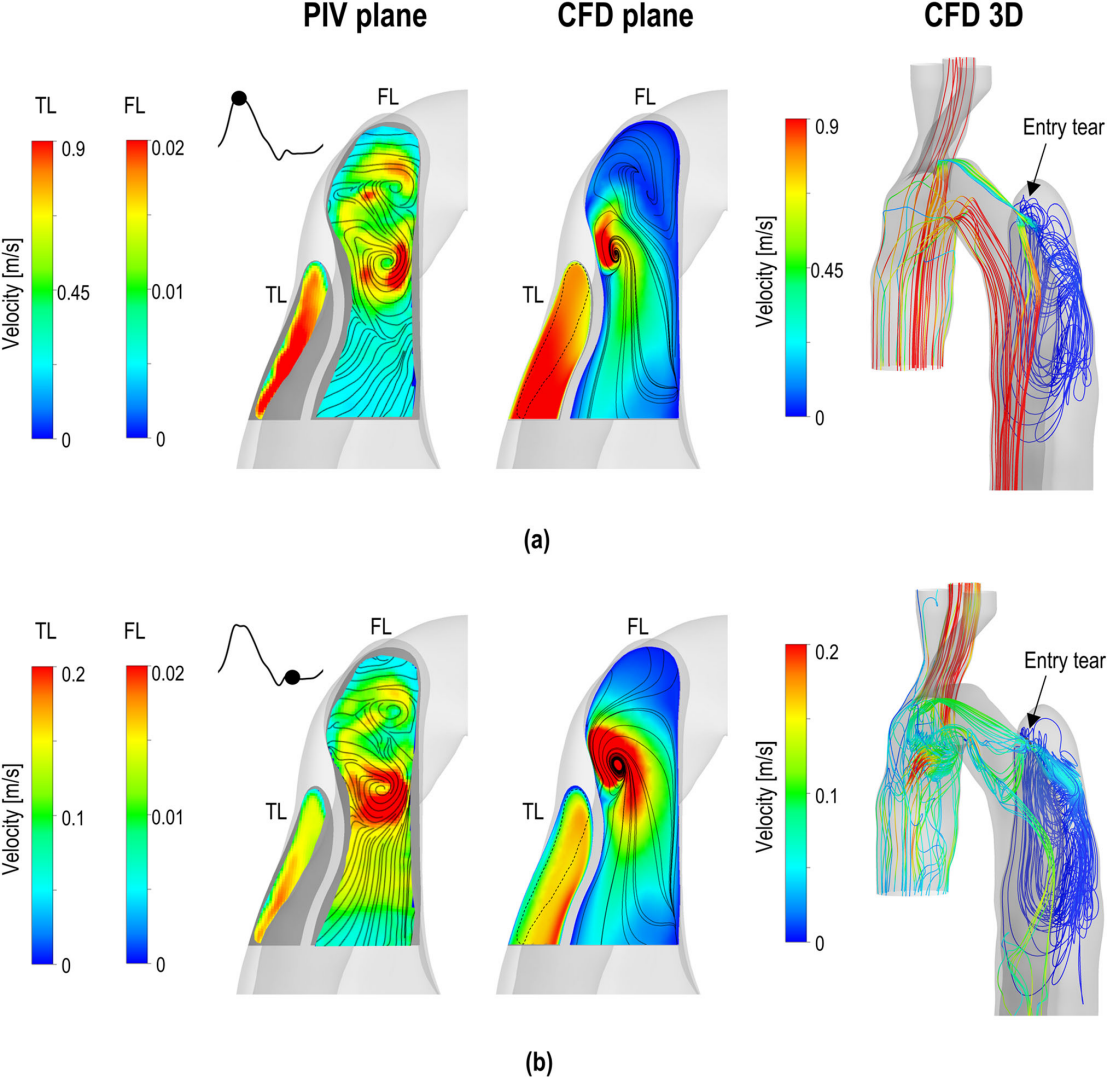
Comparison between experimental PIV-derived phase-averaged velocity magnitude fields (left column) and corresponding numerical predictions (middle column) in the proximal part of the true (TL) and false (FL) lumen. The 3D streamlines obtained with the CFD model are shown on the right. The comparison is shown at (a) peak systole and (b) early diastole. It should be noted that the colour scales between the TL and FL differ for clarity purposes.

Figure 7 shows a comparison between PIV-derived velocity fields and corresponding numerical results in the proximal part of the TL and FL. The increase of the velocity peak value due to the narrowing of the TL can be observed in both PIV and CFD results (Fig. 7a) reaching a maximum value of 0.9 m/s. Complex recirculating flow patterns are observed throughout the cardiac cycle in the FL, as illustrated by the 3D streamlines in Fig. 7. Unlike the TL, no significant flow structure and velocity magnitude variations are observed in the FL during the cardiac cycle. Interestingly, slightly higher velocity values are captured in the FL during diastole, both in the PIV and CFD results. This behaviour is due to the particular location of the imaged plane (see Fig. 2b), which does not include the entry tear and is oriented perpendicularly to its cross section. Therefore, the flow is mainly out of the imaged plane during systole, and hence the measured velocities are lower, whereas during diastole, in-plane recirculating flows develop increasing the velocities measured herein.

## Discussion

The purpose of this work was to develop a robust framework for accurate experimental and numerical haemodynamic studies of AD informed by non-invasive clinical data. The state-of-the-art models included computer-controllable pulsatile flow and finely-adjustable dynamic BCs as well as a patient-specific aortic domain.

### Aortic Dissection Haemodynamics

The experimental platform developed in this study evaluated physiological and patient-specific blood flow in an AD model with a high level of accuracy. This was achieved by properly tuning the inlet and outlet BCs of a patient-specific aortic phantom. In particular, the computer-controlled pulsatile pump—driving the flow in the mock circulation loop—produced an inlet flow rate with the characteristic features of the *in vivo* waveform acquired *via* 2D PC-MRI for the studied patient. The bespoke pump offers a higher degree of customisation and flexibility allowing the reproduction of patient-specific conditions with an unprecedented level of detail and accuracy compared to commercial systems^20^,^24^; moreover, the chosen flow configuration (i.e. piston-pump and left ventricle simulator) is more suitable to create physiological aortic flow waves, characterised by a systolic and a diastolic phase, than solutions that comprise only a displacement pump.^3^ The physical 3WK models coupled to the outlets of the phantom successfully reproduced the hydraulic impedance of the distal vasculature of the aorta, allowing for physiological pressure/flow relationships, as evidenced by the realistic waveforms acquired in the experiment. By tuning the *R* and *C* components of the 3WK for each outlet, it was possible to personalise important haemodynamic quantities, such as the flow distribution among the aortic branches and the systolic/diastolic pressure values in the aortic model. These quantities typically provide a measure of the personalisation achieved in blood flow simulations^6^; the experimentally derived flow distribution and pressures demonstrate that the same personalisation can be achieved by the developed *in vitro* platform.

High-definition PIV allowed the visualisation of the velocity fields in the aortic phantom. Axial velocity profiles at four different instants of the cardiac cycle were extracted at four selected locations in the ascending aorta/aortic arch accurately quantifying the haemodynamics therein, and reliably capturing the physiological features of the flow at all phases of the cardiac cycle. During the acceleration and peak systolic phases, the velocity profiles were skewed towards the inner curvature of the arch, in agreement with *in vitro* observations on healthy aortic phantoms.^16^ Flow separation was detected at the inner wall during the deceleration phase, while a bidirectional velocity profile with back-flow along the inner wall was measured at early diastole. This behaviour matched very well the *in vivo* measurements of blood velocity in the human ascending aorta reported by Segadal and Matre.^25^

PIV measurements in the proximal dissection demonstrated organised and high-velocity flow in the narrowed TL during the systolic phase. On the other hand, the FL was dominated by vortical and complex flow structures during both the systolic and diastolic phases. This is a common feature for AD flow as widely reported by computational studies.^9^ The absence of secondary tears in this patient led to a zero net-flow entering the FL *via* the entry tear. Consequentially, low-velocity and disturbed flow was observed in the region next to the tear throughout the cardiac cycle, while stagnant flow characterised the remaining part of the FL.

### *In Vitro* and *In Silico* Comparison

This study is the first to validate a patient-specific AD CFD model with dynamic BCs and pulsatile flow, and the first to employ PIV to investigate the haemodynamics of a personalised AD model. A comprehensive comparison between the numerical predictions and experimental results demonstrated the consistency and accuracy of the blood flow patterns obtained in the aortic domain.

Given the complexity of the pathology under investigation, the exceptional agreement achieved is a testament of the level of sophistication in our experimental platform and the potential of our combined approach. Nevertheless, small discrepancies between numerical and experimental flow fields were observed in certain regions, attributed to various, well documented,^22^ sources of error in PIV measurements, such as inaccuracies in the location of the laser sheet, construction of the physical model and near-wall refraction.

The largest differences between the experimental results and numerical predictions were found in the FL throughout the cycle and in the TL during diastole, which were characterised by disorganised and low-velocity flows. In these regions, the phase-averaged flow measurements fluctuate more, with standard deviation values comparable to the magnitude of the in-plane velocity. These standard deviation values include experimental inter-cycle variations, PIV errors and small scale turbulent motions in the flow. In addition, the accumulation of seeding particles in the areas of low-velocity or stagnant flow, such as the FL, increases laser light reflections and hence the optical noise in these regions.

As evidenced by the comparison between the PIV and CFD velocity profiles, experimental near-wall velocity gradients—necessary for WSS calculation—are prone to errors and represent a limitation on the information that can be obtained from the *in vitro* model. However, in line with the aim of this work, such limitation is complemented by the use of a validated computational model, and highlights the potential of combining experimental and numerical tools as described in the following Section.

### Clinical Relevance

This work demonstrates how *in vitro* and *in silico* tools can be developed in synergy to accurately reproduce the patient-specific haemodynamics of complex AD cases introducing a new paradigm in the treatment planning of AD or other vascular conditions.

As the recent literature shows, fluid dynamic markers, such as WSS and intra-luminal pressures, have a significant impact on the long-term progression of AD, and an informed clinical planning cannot rely only on geometric information, but must take the pathological and complicated haemodynamic environment that characterises complex AD cases into account.^6^

The value of such a synergistic approach to AD research and clinical translation cannot be underestimated. Ensuring that simulations are valid and accurate is an important step to transfer such methodologies to the clinic. The experimental tools developed in this research provide a benchmark for the validation of the *in silico* results. Once validated, CFD models can be used to estimate with high detail haemodynamic markers, such as WSS, that would be difficult to measure experimentally and impossible *in vivo*. *In silico* models can also be used for the virtual simulation of different interventional scenarios (e.g. fenestration, tear occlusions). On the other hand, experimental facilities allow the performance testing of novel and possibly personalised medical device prototypes, such as stent-grafts, that would require computationally expensive numerical models, as recently demonstrated by Birjiniuk *et al.*^3^ Realistic phantoms and flow conditions can also be used for surgical training and pre-procedural planning for AD.

### Limitations

This work was carried out under the assumption of rigid wall of the aortic phantom. This approximation was numerically investigated by Bonfanti *et al.*^4^ for the same case-study highlighting the impact of aortic compliance on the transmural pressure between the TL and FL. However, the wall displacement was less that 0.75 mm for this chronic AD, consequently, the rigid wall approximation was deemed acceptable. Nonetheless, this assumption is not always appropriate (especially for the acute stage of AD as highlighted by Baumler *et al.*^2^) and the manufacturing of phantoms with physiological mechanical properties will be addressed in future work.

In the present study a compromise had to be made with regards to the rheology of the blood mimicking fluid. *In vitro* haemodynamic works commonly employ Newtonian fluids, either water^3^ or blood analogue solutions.^20^ Since RI matching is a requirement for PIV applications, compromises must be made and $\rho$ and $\mu$ values different from those of blood have been accepted in several studies.^7^ In order to evaluate the impact of these approximations, a simulation with the rheological properties of a non-Newtonian blood model (i.e. Carrau-Yasuda viscosity model with parameters from Clarion *et al.*^10^ and $\rho$ = 1060 kg/m$^3$) was compared against the results obtained with the KCSN solution. A maximum and mean difference of 25.9 and 1.0%, respectively, was found when comparing the velocity fields at peak systole in the whole fluid domain, while a maximum difference of 3.6 and 4.3% was found between the *P* and *Q* waveforms at the boundaries. Therefore, the approximation of the working fluid was considered acceptable.

### Conclusions

A novel, personalised approach combining experimental and numerical tools informed by clinical data was introduced. For the first time, both *in vitro* and *in silico* models included an anatomically-accurate AD geometry, and physiologically-accurate and personalised pulsatile flow and dynamic BCs. As highlighted in Fig. 1, both models were developed with high integration of information at several levels, and were informed by clinical data.

The developed platform can be used to assess clinically-relevant haemodynamic markers and plan interventions as well as support the development of novel or personalised medical devices. Overall, the presented combined approach is a powerful tool for the haemodynamic investigation of AD, with a level of detail impossible to achieve hitherto. It thus represents a significant advance of the state-of-the-art on patient-specific modelling and simulation of AD haemodynamics and can be generalised and applied to other vascular conditions changing the current paradigm in clinical practise.

## Abbreviations

AD: Aortic dissection
FL: False lumen
TL: True lumen
IF: Intimal flap
CFD: Computational fluid dynamics
PIV: Particle image velocimetry
3WK: 3-Element Windkessel model
WSS: Wall shear stress
BC: Boundary condition
*R*: Resistance
*C*: Compliance
RI: Refractive index
TAWSS: Time-averaged wall shear stress
FSI: Fluid structure interaction
PC-MRI: Phase-contrast magnetic resonance imaging
CT: Computed tomography
LSA: Left subclavian artery
BT: Brachiocephalic trunk
LCC: Left common carotid
DA: Descending aorta
CO: Cardiac output
SV: Stroke volume
SST: Shear stress transport
RANS: Reynolds-averaged Navier–Stokes
